# The complete chloroplast genome sequence of *Catalpa bungei* (Bignoniaceae): a high-quality timber species from China

**DOI:** 10.1080/23802359.2020.1841581

**Published:** 2020-12-24

**Authors:** Jing Yang, Shuai Wang, Ziqi Huang, Peng Guo

**Affiliations:** a College of Landscape Architecture and Art, Henan Agricultural University, Zhengzhou, China; b College of Life Science, Henan Agricultural University, Zhengzhou, China

**Keywords:** *Catalpa bungei*, chloroplast genome, Bignoniaceae, phylogenetic analysis

## Abstract

*Catalpa bungei* is an important resource of timber, belonging to the genus *catalpa* (Bignoniaceae). In this study, we sequenced complete chloroplast (cp) genome of *C. bungei*, using a NovaSeq 6000 sequencing platform. The genome of the *C. bungei* was 152,153 bp in length, including a large single-copy region (84,910 bp), a small single-copy region (12,664 bp), and two inverted repeats regions (30,285 bp). It encodes 126 genes, including 81 protein-coding genes, 37 tRNA genes, and eight rRNA genes. Phylogenetic analyses were performed based on 15 cp genomes using the maximum likelihood (ML) method, supported that *C. bungei* was probably more closely related to *C*. *speciosa*. This study, the cp genome of *C. bungei* was assembled, which will provide more theoretical basis for determine the phylogenetic relationships of the *Catalpa* and related species.

*Catalpa bungei*, belonging to the *catalpa* genus (Bignoniaceae), is a tall deciduous tree, has been cultivated for more than two thousand years, is one of the unique precious wood species in China (Wang et al. 2007). In addition, *C. bungei* is often used as street trees due to its wide adaptability, strong environmental tolerance and high ornamental value (Wu et al. 2010). Complete chloroplast genome have been used for molecular marker and analyze the phylogenetic relationships of species (Yu et al. 2018). In this paper, the circular genome of *C. bungei* is reported, which will provide valuable genetic information for future breeding and identify relationships within the genus *Catalpa*.

French leaves of a single individual *C. bungei* were collected from Zhengzhou city (34°47′12.23″N, 113°39′26.44″E) in Henan province, China. Voucher specimen was kept in the Herbarium of the Henan Agricultural University (specimen code YJ20200512). Total genomic DNA of *C. bungei* was extracted by a protocol employing CTAB (Doyle and Doyle 1987). Subsequently, DNA purity was examined using a NanoDrop spectrophotometer and agarose gel electrophoresis. The library was sequenced by the Illumina NovaSeq 6000 platform double terminal sequencing method (pair-end 150). In total, 6.55 G of clean data was generated from the sample, used to assemble the cp genome using GetOrganelle v1.5 (Jin et al. 2018). The plastid genomes of *Catalpa ovata* (MT186670) as the reference sequence. Then CPGAVAS2 was used to annotate the sequence and the final alignment was manually corrected in Geneious Prime (Liu et al. 2012; Tillich et al. 2017). Finally, the annotated cp genome sequence of *C. bungei* was submitted to the GenBank (Accession number MT974543).

The cp genomes of *C. bungei* is 152,153 bp in length and exhibited conserved quadripartite structures. It contains a large single copy (LSC: 84,910 bp), a pair of inverted repeats (IRs: 30,285 bp), and a small single copy (SSC: 12,664 bp). The result showed that the cp genome of *C. bungei* encodes 126 unique genes, including 81 protein-coding genes, 37 tRNA genes, and eight rRNA genes. The overall GC content of the *C. bungei* genome was 38.1%, with LSC, IR, and SSC regions having 36.4%, 41.3%, and 33.6%, respectively.

In this paper, we completed an alignment of whole genome of *C. bungei* and others species using MAFFT v.7 (Kazutaka and Standley 2013). To determine the phylogenetic relationship of 13 Bignoniaceae species, a maximum likelihood (ML) tree was built using RAxML-HPC2 on XSEDE (8.2.12) with 1000 bootstrap replicates and the best fit model (GTR + F+G4) of nucleotide evolution determined by jModeltest (Darriba et al. 2012). The results showed that *C. bungei* might have a closer relationship with *Catalpa speciosa* (Figure 1). Complete cp genome information of *C. bungei* will provide valuable resources for evolutionary, taxonomic, and phylogenetic studies of Bignoniaceae.

**Figure 1.**
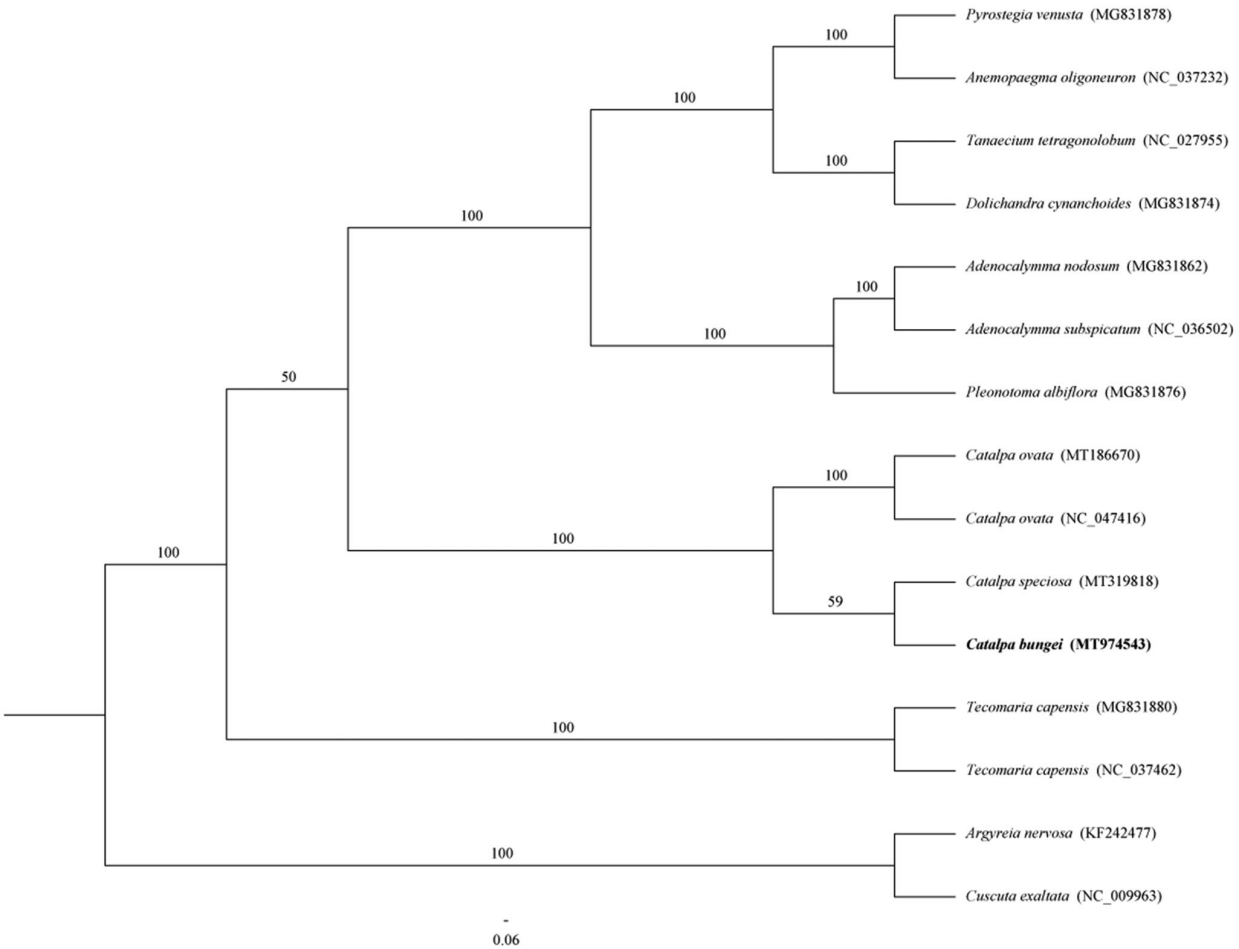
A maximum likelihood (ML) phylogenetic tree reconstruction including 15 species based on complete chloroplast genome. The position of *C. bungei* is indicated in bold.

## Data Availability

The data that support the findings of this study are openly available in the National Center for Biotechnology Information (NCBI) at https://www.ncbi.nlm.nih.gov/, reference number MT974543.

## References

[CIT0001] Darriba D, Taboada GL, Doallo R, Posada D. 2012. jModelTest 2: more models, new heuristics and parallel computing. Nat Methods. 9(8):772–772. 10.1038/nmeth.2109 PMC4594756 22847109

[CIT0002] Doyle JJ, Doyle JL. 1987. A rapid DNA isolation procedure for small quantities of fresh leaf tissue. Phytochem Bull. 19:11–15.

[CIT0003] Jin JJ, Yu WB, Yang JB, Song Y, Yi TS, Li DZ. 2018. GetOrganelle: a simple and fast pipeline for de novo assembly of a complete circular chloroplast genome using genome skimming data. BioRxiv. 2018:1–11.

[CIT0004] Kazutaka K, Standley D. 2013. MAFFT multiple sequence alignment software version 7: improvements in performance and usability. Mol Biol Evol. 30(4):772–780. 23329690 10.1093/molbev/mst010 PMC3603318

[CIT0005] Liu C, Shi L, Zhu Y, Chen H, Zhang J, Lin X, Guan X. 2012. CpGAVAS, an integrated web server for the annotation, visualization, analysis, and GenBank submission of completely sequenced chloroplast genome sequences. BMC Genomics. 13(1):715. 23256920 10.1186/1471-2164-13-715 PMC3543216

[CIT0006] Tillich M, Lehwark P, Pellizzer T, Ulbricht-Jones ES, Fischer A, Bock R, Greiner S. 2017. GeSeq - versatile and accurate annotation of organelle genomes. Nucleic Acids Res. 45(W1):W6–W11. 28486635 10.1093/nar/gkx391 PMC5570176

[CIT0007] Wang GP, Cen XC, He L, Peng FR. 2007. Effects of water stress on photosynthetic characteristics of *Catalpa bungei*. J Nanjing Forestry Univ. 31(6):57–60.

[CIT0008] Wu LH, Wang JH, Lin J. 2010. A survey of the studies on the resources of *Catalpa bungei*. J Shanghai Jiaotong Univ. 28(1):91–96.

[CIT0009] Yu SX, Gadagkar SR, Potter D, Xu DX, Zhang M, Li ZY. 2018. Phylogeny of *Spiraea* (Rosaceae) based on plastid and nuclear molecular data: implications for morphological character evolution and systematics. Perspect Plant Ecol Evol Syst. 34:109–119.

